# Effect of post-cessation hyperglycemia on cardiovascular disease and mortality among middle-aged men: an eight-year longitudinal study

**DOI:** 10.1038/s41598-017-16378-2

**Published:** 2017-11-22

**Authors:** Seulggie Choi, Kyuwoong Kim, Jooyoung Chang, Sung Min Kim, Hye-Yeon Koo, Ji-Hye Jun, Mi Hee Cho, Kiheon Lee, Sang Min Park

**Affiliations:** 1 Department of Biomedical Sciences, Seoul National University Graduate School, Seoul, Korea; 2 College of Medicine, Seoul National University, Seoul, Korea; 3 Department of Family Medicine, Seoul National University Bundang Hospital, Seongnam, Korea; 4 Department of Family Medicine, Seoul National University Hospital, Seoul, Korea

## Abstract

Smoking cessation reduces the risk of cardiovascular disease (CVD), but also elevates fasting serum glucose (FSG) levels. The effect of post-cessation hyperglycemia on cardiovascular disease is unknown. The study population consisted of 127,066 men without type 2 diabetes from the Korean National Health Insurance System – Health Screening Cohort database. Change in smoking habits and FSG was determined by the difference in smoking status and FSG levels from the first (2002 and 2003) and second (2004 and 2005) health examinations. Continual smokers, quitters, ex-smokers, and never smokers were stratified according to FSG elevation. The study participants were followed-up for CVD and CVD-related death from 2006 to 2013. Compared to continual smokers, quitters had decreased risk of CVD among those without FSG elevation (hazard ratio, HR, 0.76, 95% confidence interval, CI, 0.66–0.86) and with FSG elevation (HR 0.83, 95% CI 0.72–0.96). Similarly, quitters had a tendency towards reduced risk of CVD-related death among those without FSG elevation (HR 0.74, 95% CI 0.51–1.09) and with FSG elevation (HR 0.68, 95% CI 0.46–1.03). Post-cessation hyperglycemia did not attenuate the beneficiary risk-reducing effects of quitting on CVD and CVD-related death.

## Introduction

Smoking is considered one of the most important causes of preventable death worldwide^1^. In the Unites States alone, smoking is estimated to account for approximately 480,000 deaths, or 20 percent of deaths annually^2^. This is due to the fact that smoking causes a number of serious illnesses, including cardiovascular disease (CVD)^3^. While smoking cessation reduces the risk of CVD, quitting also has temporary side effects that may attenuate this beneficiary risk-reducing effect on CVD. Multiple studies have shown that smoking cessation temporarily increases the risk of type 2 diabetes, with quitters having a 14 to 54% increased risk of diabetes within the first two to three years^4–9^. This increase in type 2 diabetes risk may be due to the deterioration of glycemic control following smoking cessation, reflected by increased fasting serum glucose (FSG) or HbA1c levels after quitting depicted in previous studies^9–12^.

Elevation of FSG is positively associated with the risk of cardiovascular disease and CVD-related death^13–15^. Tanne and colleagues revealed that there was a positive relationship between FSG and cardiovascular disease^15^. Similarly, Wei and colleagues have shown that the risk of CVD-related mortality increased in rising FSG levels in a dose-responsive manner^14^. Finally, Barr and colleagues revealed that for every incremental 12.6 mg/dL increase in FSG levels among those above FSG of 90 mg/dL, there was a 30% increase in the risk of CVD-related mortality (hazard ratio, HR, 1.3, 95% confidence interval, CI, 1.1–1.4)^13^.

While smoking cessation reduces the risk of CVD and CVD-related death, whether or not post-cessation hyperglycemia attenuates the risk-reducing effect of quitting on CVD has not yet been explored. Therefore, we aimed to determine the effect smoking cessation with and without FSG elevation on the risk of CVD and CVD-related death using the Korean National Health Insurance System – National Health Screening Cohort (NHIS-HealS) database.

## Results

Table 1 shows the descriptive characteristics of the study population. The mean values of initial FSG for continual smokers, quitters, ex-smokers, and never smokers are 91.4 mg/dL, 91.2 mg/dL, 92.3 mg/dL, and 91.6 mg/dL, respectively. The mean values of change in FSG between the second and first health examinations were 3.4 mg/dL, 3.9 mg/dL, 2.6 mg/dL, and 2.6 mg/dL for continual smokers, quitters, ex-smokers, and never smokers, respectively. The change in FSG for quitters was significantly greater than that for continual smokers, which was determined by the analysis of variance (ANOVA) method (*p*-value < 0.01).

**Table 1:** Descriptive characteristics of the study population.

	Continual smokers	Quitters	Ex-smokers	Never smokers
Number of people	43,627	13,513	24,945	44,981
Age, years, mean (SD)	51.7 (7.8)	52.5 (8.2)	53.6 (8.8)	54.6 (9.0)
Fasting serum glucose, mg/dL, mean (SD)				
First examination	91.4 (13.1)	91.2 (12.9)	92.3 (12.7)	91.6 (12.8)
Second examination	94.8 (18.7)	95.1 (18.3)	95.0 (16.8)	94.3 (16.8)
Change	3.4 (19.9)	3.9 (19.3)	2.6 (17.6)	2.6 (17.9)
Socioeconomic status, N (%)				
First quartile (highest)	16,065 (36.8)	5,730 (42.4)	12,303 (49.3)	19,718 (43.8)
Second quartile	13,932 (31.9)	4,000 (29.6)	6,914 (27.7)	13,035 (29.0)
Third quartile	8,280 (19.0)	2,314 (17.1)	3,633 (14.6)	7,636 (17.0)
Fourth quartile (lowest)	5,350 (12.3)	1,469 (10.9)	2,095 (8.4)	4,592 (10.2)
Physical activity, times per week, N (%)				
None	19,930 (45.7)	6,988 (51.7)	9,025 (36.2)	19,295 (42.9)
1–2	15,407 (35.3)	3,785 (28.0)	8,659 (34.7)	14,224 (31.6)
3–4	4,984 (11.4)	1,689 (12.5)	4,265 (17.1)	6,305 (14.0)
5–7	3,306 (7.6)	1,051 (7.8)	2,996 (12.0)	5,157 (11.5)
Alcohol consumption, drinks per week, N (%)				
None	9,513 (21.8)	6,630 (49.1)	7,865 (31.5)	22,207 (49.4)
1–2	23,260 (53.3)	5,251 (38.9)	12,829 (51.4)	17,932 (39.9)
≥3	10,854 (24.9)	1,632 (12.1)	4,251 (17.0)	4,842 (10.8)
Body mass index, kg/m^2^, mean (SD)	23.6 (2.8)	24.0 (2.8)	24.2 (2.7)	24.0 (2.7)
Systolic blood pressure, mmHg, mean (SD)	126.6 (16.3)	127.1 (16.4)	127.6 (15.9)	127.8 (16.4)
Diastolic blood pressure, mmHg, mean (SD)	80.2 (10.9)	80.4 (10.9)	80.5 (10.7)	80.6 (10.8)
Total cholesterol, mg/dL, mean (SD)	196.6 (36.3)	198.5 (36.6)	197.5 (35.3)	194.5 (34.9)
Charlson comorbidity index, %				
0	20,217 (46.3)	5,779 (42.8)	9,756 (39.1)	17,754 (39.5)
1	13,349 (30.6)	4,109 (30.4)	8,053 (32.3)	14,094 (31.3)
≥2	10,061 (23.1)	3,625 (26.8)	7,136 (28.6)	13,133 (29.2)

Acronyms: SD, standard deviation; N, number of people.

**Figure 1:**
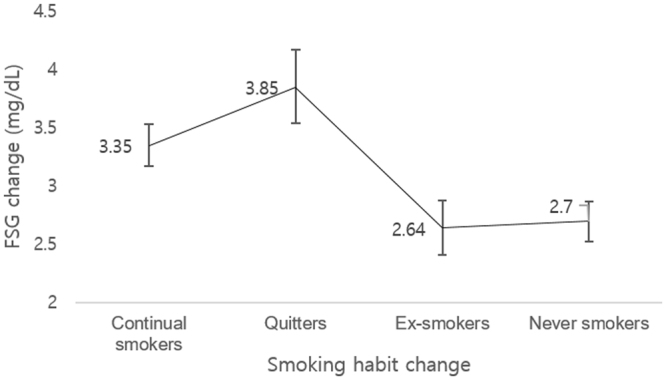
Effect of smoking habit change on change in fasting serum glucose. The adjusted mean values of fasting serum glucose was determined for continual smokers, quitters, ex-smokers, and never smokers. Adjusted means of change in fasting serum glucose calculated by linear regression analysis with adjustments for age, socioeconomic status, physical activity, alcohol consumption, body mass index, blood pressure, total cholesterol, and Charlson comorbidity index Acronyms: FSG, fasting serum glucose.

Figure 1 depicts the effect of smoking habit change on change in FSG levels. The adjusted mean values (95% CI) for FSG change for continual smokers, quitters, ex-smokers, and never smokers were 3.35 (2.17–3.53) mg/dL, 3.85 (3.54–4.17) mg/dL, 2.64 (2.41–2.87) mg/dL, and 2.70 (2.52–2.87) mg/dL, respectively. Compared to continual smokers, the change in FSG levels was significantly greater among quitters (*p* value 0.008). Compared to never smokers, ex-smokers and never smokers had lower changes in FSG levels (*p* values < 0.001).

The association between smoking habit change with and without FSG elevation on CVD is shown in Table 2. Compared to continual smokers, quitters had reduced risk of CVD among those without FSG elevation (HR 0.76, 95% CI 0.66–0.86) and with FSG elevation (HR 0.83, 95% CI 0.72–0.96). Both quitters without FSG elevation and without FSG elevation had reduced risk myocardial infarction (HR 0.43, 95% CI 0.31–0.60 and HR 0.60, 95% CI 0.43–0.83, respectively). Quitters tended to have decreased risk of total stroke among those without FSG elevation (HR 0.87, 95% CI 0.74–1.01) and with FSG elevation (HR 0.91, 95% CI 0.77–1.06).

**Table 2:** Effect of smoking habit change with and without fasting serum glucose elevation on cardiovascular disease.

		Continual smokers	Quitters	Ex-smokers	Never smokers
Cardiovascular disease	Without FSG elevation*				
	Events	1,057	278	483	927
	Person-years	190,574	57,650	112,965	203,865
	aHR (95% CI)	1.00 (reference)	0.76 (0.66–0.86)	0.62 (0.56–0.69)	0.59 (0.54–0.65)
	With FSG elevation				
	Events	817	253	369	678
	Person-years	143,586	46,187	79,728	144,051
	aHR (95% CI)	1.00 (reference)	0.83 (0.72–0.96)	0.62 (0.55–0.71)	0.58 (0.52–0.64)
Myocardial infarction	Without FSG elevation				
	Events	251	44	77	130
	Person-years	193,142	58,388	114,212	206,254
	aHR (95% CI)	1.00 (reference)	0.43 (0.31–0.60)	0.40 (0.31–0.52)	0.32 (0.25–0.40)
	With FSG elevation				
	Events	193	47	57	88
	Person-years	145,645	46,817	80,692	145,969
	aHR (95% CI)	1.00 (reference)	0.60 (0.43–0.83)	0.41 (0.30–0.55)	0.31 (0.24–0.41)
Total stroke	Without FSG elevation				
	Events	814	239	415	806
	Person-years	191,437	57,795	113,177	204,259
	aHR (95% CI)	1.00 (reference)	0.87 (0.75–1.01)	0.71 (0.64–0.80)	0.69 (0.62–0.76)
	With FSG elevation				
	Events	638	211	313	602
	Person-years	144,182	46,308	79,949	144,340
	aHR (95% CI)	1.00 (reference)	0.91 (0.77–1.06)	0.68 (0.59–0.78)	0.66 (0.59–0.74)
Ischemic stroke	Without FSG elevation				
	Events	487	144	245	456
	Person-years	192,442	58,103	113,660	205,235
	aHR (95% CI)	1.00 (reference)	0.86 (0.71–1.04)	0.67 (0.57–0.79)	0.62 (0.54–0.71)
	With FSG elevation				
	Events	390	125	170	332
	Person-years	144,912	46,567	80,326	145,212
	aHR (95% CI)	1.00 (reference)	0.85 (0.69–1.04)	0.57 (0.48–0.69)	0.56 (0.48–0.65)
Hemorrhagic stroke	Without FSG elevation				
	Events	170	48	65	160
	Person-years	193,445	58,364	114,236	206,189
	aHR (95% CI)	1.00 (reference)	0.91 (0.65–1.26)	0.59 (0.44–0.80)	0.76 (0.60–0.96)
	With FSG elevation				
	Events	120	40	56	134
	Person-years	145,916	46,825	80,764	145,812
	aHR (95% CI)	1.00 (reference)	1.02 (0.71–1.47)	0.76 (0.55–1.05)	0.96 (0.74–1.24)

*FSG elevation defined by an elevation of fasting serum glucose of more than the upper limit of 95% confidence interval for FSG change among quitters (>4.17mg/dL)

Hazard ratio calculated by Cox proportional hazards regression analysis with adjustments for age, socioeconomic status, physical activity, alcohol consumption, body mass index, baseline fasting serum glucose, blood pressure, total cholesterol, and Charlson comorbidity index

Acronyms: FSG, fasting serum glucose; aHR, adjusted hazard ratio; CI, confidence interval.

The effect of smoking habit change with and without FSG elevation on CVD-related death is shown in Table 3. Compared to continual smokers, quitters tended to have reduced risk of CVD-related death among those without FSG elevation (HR 0.74, 95% CI 0.51–1.09) and with FSG elevation (HR 0.68, 95% CI 0.46–1.03). Quitters among those with FSG elevation had decreased risk of myocardial infarction-related death (HR 0.26, 95% CI 0.11–0.62), compared to continual smokers. Both quitters with and without FSG elevation did not have reduced risk of total stroke-related death (HR 0.80, 95% CI 0.47–1.35 and HR 1.11, 95% CI 0.69–1.80, respectively). Sensitivity analyses on the effect of smoking habit change with and without FSG elevation on CVD and CVD-related death after excluding participants with events occurring within the first one to four years of follow-up are shown in Table 4. The effect of post-cessation hyperglycemia on CVD and CVD-related death was not altered after excluding events that occurred within the first one to four years of follow-up. Compared to continual smokers, quitters had reduced risk of CVD among those with and without FSG elevation after excluding participants diagnosed with CVD within the first four years of follow-up.

**Table 3:** Effect of smoking habit change with and without fasting serum glucose elevation on cardiovascular disease-related death.

		Continual smokers	Quitters	Ex-smokers	Never smokers
Cardiovascular disease-related death	Without FSG elevation*				
	Events	133	34	72	127
	Person-years	194,009	58,546	114,444	206,670
	aHR (95% CI)	1.00 (reference)	0.74 (0.51–1.09)	0.68 (0.50–0.91)	0.59 (0.46–0.76)
	With FSG elevation				
	Events	120	30	42	100
	Person-years	146,281	46,952	80,915	146,282
	aHR (95% CI)	1.00 (reference)	0.68 (0.46–1.03)	0.49 (0.34–0.70)	0.58 (0.44–0.77)
Myocardial infarction-related death	Without FSG elevation				
	Events	68	16	33	51
	Person-years	194,009	58,546	114,444	206,670
	aHR (95% CI)	1.00 (reference)	0.68 (0.39–1.18)	0.67 (0.44–1.03)	0.50 (0.34–0.73)
	With FSG elevation				
	Events	59	6	20	29
	Person-years	146,281	46,952	80,915	146,282
	aHR (95% CI)	1.00 (reference)	0.26 (0.11–0.62)	0.50 (0.30–0.84)	0.36 (0.22–0.57)
Total stroke-related death	Without FSG elevation				
	Events	65	18	39	76
	Person-years	194,009	58,546	114,444	206,670
	aHR (95% CI)	1.00 (reference)	0.80 (0.47–1.35)	0.69 (0.46–1.03)	0.68 (0.48–0.96)
	With FSG elevation				
	Events	61	24	22	71
	Person-years	146,281	46,952	80,915	146,282
	aHR (95% CI)	1.00 (reference)	1.11 (0.69–1.80)	0.48 (0.29–0.79)	0.79 (0.55–1.13)
Ischemic stroke-related death	Without FSG elevation				
	Events	19	5	15	32
	Person-years	194,009	58,546	114,444	206,670
	aHR (95% CI)	1.00 (reference)	0.67 (0.25–1.82)	0.76 (0.38–1.53)	0.82 (0.45–1.50)
	With FSG elevation				
	Events	14	10	5	524
	Person-years	146,281	46,952	80,915	146,282
	aHR (95% CI)	1.00 (reference)	2.02 (0.88–2.64)	0.48 (0.17–1.35)	1.16 (0.58–2.33)
Hemorrhagic stroke-related death	Without FSG elevation				
	Events	25	2	15	22
	Person-years	194,009	58,546	114,444	206,670
	aHR (95% CI)	1.00 (reference)	—	0.81 (0.42–1.58)	0.59 (0.32–1.10)
	With FSG elevation				
	Events	25	13	10	20
	Person-years	146,281	46,952	80,915	146,282
	aHR (95% CI)	1.00 (reference)	1.28 (0.79–3.15)	0.61 (0.29–1.30)	0.63 (0.34–1.18)

*FSG elevation defined by an elevation of fasting serum glucose of more than the upper limit of 95% confidence interval for FSG change among quitters (>4.17 mg/dL)

Hazard ratio calculated by Cox proportional hazards regression analysis with adjustments for age, socioeconomic status, physical activity, alcohol consumption, body mass index, baseline fasting serum glucose, blood pressure, total cholesterol, and Charlson comorbidity index

Acronyms: FSG, fasting serum glucose; aHR, adjusted hazard ratio; CI, confidence interval

Blank cells indicate no reliable values of hazard ratios and 95% confidence intervals due to the small number of events.

**Table 4:** Sensitivity analysis of the effect of smoking habit change with and without fasting serum glucose elevation on cardiovascular disease and cardiovascular disease-related death after excluding participants with events occurring within the first one to four years of follow-up.

	Exclusion period		Continual smokers	Quitters	Ex-smokers	Never smokers
			aHR (95% CI)			
Cardiovascular disease	One year	Without FSG elevation*	1.00 (reference)	0.77 (0.67–0.89)	0.64 (0.57–0.72)	0.61 (0.55–0.67)
		With FSG elevation	1.00 (reference)	0.85 (0.73–0.98)	0.62 (0.54–0.70)	0.56 (0.50–0.63)
	Two years	Without FSG elevation	1.00 (reference)	0.82 (0.70–0.96)	0.60 (0.52–0.69)	0.56 (0.50–0.63)
		With FSG elevation	1.00 (reference)	0.78 (0.67–0.91)	0.65 (0.57–0.73)	0.61 (0.55–0.68)
	Three years	Without FSG elevation	1.00 (reference)	0.82 (0.70–0.96)	0.60 (0.52–0.69)	0.56 (0.50–0.63)
		With FSG elevation	1.00 (reference)	0.79 (0.67–0.92)	0.65 (0.57–0.75)	0.64 (0.57–0.71)
	Four years	Without FSG elevation	1.00 (reference)	0.83 (0.70–0.99)	0.61 (0.52–0.71)	0.55 (0.48–0.63)
		With FSG elevation	1.00 (reference)	0.83 (0.69–0.99)	0.68 (0.58–0.78)	0.63 (0.56–0.72)
Cardiovascular disease-related death	One year	Without FSG elevation	1.00 (reference)	0.81 (0.54–1.21)	0.74 (0.54–1.00)	0.64 (0.49–0.85)
		With FSG elevation	1.00 (reference)	0.72 (0.47–1.10)	0.49 (0.34–0.72)	0.62 (0.46–0.83)
	Two years	Without FSG elevation	1.00 (reference)	0.85 (0.55–1.29)	0.77 (0.56–1.08)	0.69 (0.52–0.92)
		With FSG elevation	1.00 (reference)	0.74 (0.47–1.16)	0.52 (0.34–0.77)	0.64 (0.47–0.88)
	Three years	Without FSG elevation	1.00 (reference)	0.85 (0.54–1.35)	0.83 (0.58–1.17)	0.67 (0.49–0.92)
		With FSG elevation	1.00 (reference)	0.77 (0.47–1.26)	0.54 (0.35–0.84)	0.72 (0.51–1.01)
	Four years	Without FSG elevation	1.00 (reference)	0.80 (0.48–1.34)	0.79 (0.54–1.16)	0.66 (0.47–0.93)
		With FSG elevation	1.00 (reference)	0.72 (0.42–1.23)	0.48 (0.29–0.78)	0.72 (0.50–1.03)

*FSG elevation defined by an elevation of fasting serum glucose of more than the upper limit of 95% confidence interval for FSG change among quitters (>4.17 mg/dL)

Hazard ratio calculated by Cox proportional hazards regression analysis with adjustments for age, socioeconomic status, physical activity, alcohol consumption, body mass index, baseline fasting serum glucose, blood pressure, total cholesterol, and Charlson comorbidity index

Acronyms: FSG, fasting serum glucose; aHR, adjusted hazard ratio; CI, confidence interval.

The effect of smoking habit change on CVD without stratification by FSG elevation is shown in Supplemental Table S1. Compared to continual smokers, quitters (HR 0.79, 95% CI 0.72–0.87), ex-smokers (HR 0.62, 95% CI 0.57–0.68), and never smokers (HR 0.59, 95% CI 0.55–0.63) all had reduced risk CVD. Quitters, ex-smokers, and never smokers all had decreased risk of CVD-related death (HR 0.71, 95% CI 0.54–0.94, HR 0.59, 95% CI 0.47–0.74, and HR 0.58, 95% CI 0.48–0.70, respectively), compared to continual smokers.

## Discussion

In this large-scale cohort study of over 120,000 men without type 2 diabetes, we have shown that post-cessation hyperglycemia does not attenuate the beneficiary risk-reducing effects of smoking cessation on CVD and CVD-related death. To our knowledge, this is the first study to investigate the effect of smoking habit change with and without FSG elevation on CVD and CVD-related death.

Similar to our results, several previous studies have shown that smoking cessation is associated with elevated FSG levels. Two separate studies investigating the changes in FSG levels after quitting revealed net gains of 8.1 mg/dL^12^ and 5.4 mg/dL^11^ of FSG after smoking cessation. Although the magnitude of FSG elevation upon smoking cessation in our study was smaller compared to those in previous studies, this may be explained by the fact that our study population was limited to non-diabetic male patients. While it has been suggested that post-cessation hyperglycemia was due to post-cessation weight gain, two recent studies have shown that this hyperglycemic state persisted even after adjusting for weight gain following smoking cessation^9,10^. The exact mechanisms causing post-cessation hyperglycemia is yet unclear and merit further investigation.

Multiple studies support the positive relationship between FSG levels and the risk of CVD^13–16^. Experimental studies have revealed that hyperglycemia disrupts the function of normal endothelial cells, contributes to the formation of atherosclerotic plaques, and accelerate the rupture of plaques, leading to thrombosis of the vessels^16^. Furthermore, previous epidemiological studies have shown that hyperglycemia is associated with arterial stiffness^17^, intima-media thickening of the endothelial wall, and endothelial dysfunction of the arterial wall^18^. While FSG elevation may increase the risk of CVD through these mechanisms, the results from our study suggest that the benefit of smoking cessation outweighs the potential deleterious effect of post-cessation hyperglycemia on the risk of CVD.

A large body of evidence indicates that smoking increases the risk of CVD. Recently, a meta-analysis of 25 cohorts and 503,905 participants revealed that smokers had elevated risk of CVD (HR 2.07, 95% CI 1.82–2.36) compared to never smokers^3^. Carbon monoxide from cigarette smoke is known to bind to hemoglobin, resulting in the inhibition of oxygen binding. The consequent chronic hypoxic state leads to increased blood viscosity, ultimately resulting in a hypercoagulable state due to smoking^19^. Furthermore, it has been shown that smokers have elevated levels of circulating fibrinogen^20^, which may promote thrombus formation synergistically with increased blood viscosity. Smoking also increases the formation of hydrogen peroxide and superoxide^21^, which could lead to increased thrombogenesis by inducing oxidative stress and cellular damage^22^. The decrease in these pathophysiological processes upon smoking cessation may have contributed to the reduction of CVD risk among quitters.

Quitting was more beneficial in reducing the risk of myocardial infarction compared total stroke, regardless of FSG elevation. This may explained by the fact that myocardial infarction and stroke, particularly hemorrhagic stroke, have distinct pathophysiological mechanisms. While myocardial infarction is the result of vessel occlusion, rupture of the vessel wall is the overriding mechanism for hemorrhagic stroke. Particularly, as one of the most important consequences of smoking is the development of a hypercoagulable state, it is reasonable to assume that smoking cessation may have a greater beneficiary effect on preventing myocardial infarction where hypercoagulability would elevate the risk of thrombogenesis. This is supported in a previous study showing that smoking cessation led to decreased risk of myocardial infarction (HR 0.43, 95% CI 0.34–0.53) but not hemorrhagic stroke (HR 0.82, 95% CI 0.64–1.06) compared to continual smokers^23^.

Several limitations need to be considered when interpreting the results of our study. First, as smoking status was determined by a questionnaire, it may not exactly reflect the actual smoking status of the study participant. Second, since smoking status was not followed-up after the second health examination, we do not know how the smoking status was altered afterwards. Third, as smoking habit change and FSG elevation were both measured during the same period, we cannot determine that smoking cessation preceded FSG elevation with certainty. However, multiple previous longitudinal studies have shown that quitting is associated with hyperglycemia^11,12^. Fourth, we used FSG levels in order to assess glycemic control as HbA1c values were not available. Further studies using HbA1c levels to determine the effect of post-cessation hyperglycemia on cardiovascular disease and CVD-related mortality are needed. However, in large-scale population studies, FSG has been used as a marker for glycemic control in multiple studies^11,12,16^. Fifth, the study population was limited to men aged 40 years or more. Further investigations studying the effect of post-cessation hyperglycemia on cardiovascular disease among younger aged adults and women are needed. Finally, myocardial infarction and total stroke were included in CVD, leaving out other cardiovascular events such as peripheral arterial disease that are known to be affected by smoking and hyperglycemia. While the focus of this study on major cardiovascular events, future studies investigating the association between post-cessation hyperglycemia and other cardiovascular events are needed.

Despite these limitations, the study has a number of strengths. To our knowledge, this is the first study to examine the effects of elevated FSG after smoking cessation on the risk of CVD and CVD-related death. Furthermore, the large study population, relatively long follow-up period, extensive number of covariates considered and accurate health records from the claims database add to the reliability of our results. Finally, extensive sensitivity analyses revealed that the risk reduction in CVD and CVD-related death upon smoking cessation regardless of FSG elevation was preserved after excluding events that occurred within the first four years of follow-up.

In conclusion, smoking cessation was associated with reduced risk of CVD and CVD-related death regardless of FSG elevation. Particularly, smoking cessation may be beneficial in reducing the risk of myocardial infarction regardless of FSG elevation. Smokers should be encouraged to quit in order to benefit from reduced risk of CVD and CVD-related death despite post-cessation hyperglycemia.

## Methods

### Participants

The study population was derived from the NHIS-HealS research database. The enrollment rate for the NHIS is 97% due to the fact that health insurance enrollment, provided by NHIS, became mandatory for all Korean citizens since the National Health Insurance Act in 1989. All enrollees who turn 40 years old are required to undergo biannual health examinations. Based on the data from these health examinations, the NHIS constructs datasets by a simple random sampling method and provides data on sociodemographics, hospital visits, and laboratory and other clinical data for research purposes. The data used in this study is directly available via the NHIS database registration system. Various fields of research have used this NHIS database for multiple epidemiological studies, and its validity has been described in detail elsewhere^24,25^.

A total of 176,422 men who participated in health examinations between the first (2002 to 2003) and second (2002 to 2003) periods with available smoking status and FSG values were recruited. Among them, we excluded 30,979 men who were diagnosed with diabetes during the first health examination period. Furthermore, 8,939 men who were diagnosed with cardiovascular disease and 341 men who passed away before the index date of 1 January 2006 were excluded. Then, 8,609 men with unwanted smoking status (new smokers and relapsers), 215 men with extreme outliers (lower and upper 0.05% of distribution)^26^ for FSG, and 1,481 men with missing values on covariates. Ultimately, the final study population consisted of 127,066 participants.

The Seoul National University Hospital Institutional Review Board (IRB) approved this study (IRB number: X-1701/378–902) and waived the requirement for informed consent from study participants as the NHIS database is anonymized in adherence to strict confidentiality guidelines. All experiments were performed in accordance with the relevant guidelines and regulations.

### Key variables

Study participants were grouped according to the change in smoking habit, determined by a self-reported questionnaire, between the first and second health examinations. Participants were divided into continual smokers, quitters, ex-smokers, and never smokers. Fasting serum glucose, which was measured by a blood exam during each health examination, was used to determine FSG elevation. FSG elevation was defined as an increase of more than the upper limit of the 95% confidence interval for the adjusted mean value of FSG change among quitters (4.17 mg/dL). The participants were divided into those without FSG elevation and those with FSG elevation.

Within the NHIS database, hospital admission records were used to identify cases of CVD and CVD-related death. Cardiovascular disease was defined using the codes from the Tenth Revision of International Classification of Diseases (ICD-10) from the World Health Organization. Myocardial infarction (ICD-10 codes: I21, I22, I23, I24) and total stroke (ICD-10 codes: I60, I61, I62, I63, I64, I65, I66, I67, I68, I69) were included in CVD. Total stroke was further divided into ischemic stroke (ICD-10 codes: I63) and hemorrhagic stroke (ICD-10 codes: I60, I61, I62). We defined an event of CVD as cases with two or more days of hospital admission with at least one of the ICD-10 codes pertaining to CVD. Mortality was determined by participants with a death date between 1 January 2006 and 31 December 2013. Among those with a death date, cause-specific mortality was identified by the cause of death using the ICD-10 code.

### Statistical analysis

All participants were followed-up starting 1 January 2006 and ended at the date of diagnosis of CVD, date of death, or 31 December 2013, whichever came first. Age (continuous, years), socioeconomic status (SES, categorical, first, second, third, and fourth quartiles), physical activity (categorical, none, 1–2, 3–4, and 5–7 times per week), alcohol consumption (categorical, none, 1–2, and 3 or more times), body mass index (BMI, continuous, kg/m^2^), systolic blood pressure (continuous, mmHg), diastolic blood pressure (continuous, mmHg), total cholesterol (continuous, mg/dL), baseline fasting serum glucose (continuous, mmHg), and Charlson comorbidity index (CCI, categorical, 0, 1, or 2 or more) were considered potential confounding covariates and extracted between 2002 and 2005. SES was categorized according to each patient’s insurance premium status. CCI was calculated using the ICD-10 code diagnoses for major comorbidities between 2002 and 2005 based on the claims database. The algorithm for the calculation of CCI by ICD-10 codes was adapted from another study^27^.

The adjusted mean values for FSG change for continual smokers, quitters, ex-smokers, and never smokers were calculated by linear regression analysis. We conducted Cox proportional hazards regression analyses to obtain the hazard ratios (HRs) and 95% confidence intervals (CI) of the risk of CVD and CVD-related mortality according to smoking habit change with and without FSG elevation. In all analyses, continual smokers were used as references. The multivariate-adjusted analysis was adjusted for age, BMI, blood pressure, baseline FSG, total cholesterol, physical activity, smoking status, drinking habit, SES, and CCI. To minimize the possibility of reverse-causality, in which smokers quit due to worsening health conditions that may elevate the risk of CVD, sensitivity analysis of the effect of smoking habit change with and without FSG elevation on CVD and CVD-related death were conducted by excluding participants with events occurring within the first one to four years of follow-up.

Statistical significance was defined as a *p*-value of less than 0.05 in a two-sided manner. All data collection and statistical analyses conducted in this study were done with SAS 9.3 (SAS Institute, Cary, NC, USA) and STATA 13.0 (StataCorp LP, College Station, TX, USA), respectively.

## Electronic supplementary material

Supplemental Table S1
