# Supplementary material for: Effect of post-cessation hyperglycemia on cardiovascular disease and mortality among middle-aged men: an eight-year longitudinal study
Source: Sci Rep. 2017 Nov 22;7:16085. doi: 10.1038/s41598-017-16378-2 (PMC5700198; doi:10.1038/s41598-017-16378-2)
Supplement: Supplementary file 1 — Supplemental Table S1 [file 41598_2017_16378_MOESM1_ESM.doc]

**Effect of post-cessation hyperglycemia on cardiovascular disease and mortality among middle-aged men: an eight-year longitudinal study**

Seulggie Choi^1^, Kyuwoong Kim^1^, Jooyoung Chang^2^, Sung Min Kim^1^, Hye-Yeon Koo^3^, Ji-Hye Jun^3^, Mi Hee Cho^4^, Kiheon Lee^3,4^, and Sang Min Park^1,4^

^1^Department of Biomedical Sciences, Seoul National University College of Medicine, Seoul, Korea

^2^College of Medicine, Seoul National University, Seoul, Korea

^3^Department of Family Medicine, Seoul National University Bundang Hospital, Seongnam, Korea

^4^Department of Family Medicine, College of Medicine, Seoul National University, Seoul, Korea

Correspondence should be addressed to Kiheon Lee and Sang Min Park:

Kiheon Lee, Department of Family Medicine, Seoul National University College of Medicine and Seoul National University Bundang Hospital, 82 Gumi-ro 173 beon-gil, Bundang-gu, Seongnam, Korea

Tel.: +82-31-787-7801 Fax: +82-31-787-4834

e-mail: keyhoney@gmail.com

Sang Min Park, Department of Family Medicine and Biomedical Sciences, College of Medicine, Seoul National University, 101 Daehak-ro, Jongno-gu, Seoul, Korea

Tel.: +82-2-2072-3331 Fax: +82-2-766-3276

e-mail: smpark.snuh@gmail.com

**Supplemental Table S1.** Effect of smoking habit change on cardiovascular disease and cardiovascular disease-related death.

|  | Continual smokers | Quitters | Ex-smokers | Never smokers |
| --- | --- | --- | --- | --- |
| Cardiovascular disease |  |  |  |  |
| Events | 1,874 | 531 | 852 | 1,605 |
| Person-years | 143,586 | 190,584 | 46,187 | 57,560 |
| aHR (95% CI) | 1.00 (reference) | 0.79 (0.72-0.87) | 0.62 (0.57-0.68) | 0.59 (0.55-0.63) |
| Cardiovascular disease-related death |  |  |  |  |
| Events | 253 | 64 | 114 | 227 |
| Person-years | 146,281 | 194,009 | 46,952 | 58,546 |
| aHR (95% CI) | 1.00 (reference) | 0.71 (0.54-0.94) | 0.59 (0.47-0.74) | 0.58 (0.48-0.70) |

Hazard ratio calculated by Cox proportional hazards regression analysis with adjustments for age, socioeconomic status, physical activity, alcohol consumption, body mass index, baseline fasting serum glucose, blood pressure, total cholesterol, and Charlson comorbidity index

Acronyms: aHR, adjusted hazard ratio; CI, confidence interval
